# Efficient clathrin-mediated entry of enteric adenoviruses in human duodenal cells

**DOI:** 10.1128/jvi.00770-23

**Published:** 2023-10-12

**Authors:** Miriam Becker, Dario Valter Conca, Noemi Dorma, Nitesh Mistry, Elin Hahlin, Lars Frängsmyr, Marta Bally, Niklas Arnberg, Gisa Gerold

**Affiliations:** 1 Department of Biochemistry & Research Center for Emerging Infections and Zoonoses (RIZ), University of Veterinary Medicine Hannover, Hannover, Germany; 2 Department of Clinical Microbiology, Umeå University, Umeå, Sweden; 3 Wallenberg Centre for Molecular Medicine (WCMM), Umeå University, Umeå, Sweden; 4 Molecular Infection Medicine Sweden (MIMS), Umeå University, Umeå, Sweden; International Centre for Genetic Engineering and Biotechnology, Trieste, Italy

**Keywords:** enteric adenovirus, virus entry, single particle tracking, clathrin-mediated endocytosis, electron microscopy

## Abstract

Enteric adenovirus types F40 and 41 (EAdVs) are a leading cause of diarrhea and diarrhea-associated death in young children and have recently been proposed to cause acute hepatitis in children. EAdVs have a unique capsid architecture and exhibit — unlike other human adenoviruses — a relatively strict tropism for gastrointestinal tissues with, to date, understudied infection mechanism and unknown target cells. In this study, we turn to potentially limiting host factors by comparing EAdV entry in cell lines with respiratory and intestinal origin by cellular perturbation, virus particle tracking, and transmission electron microscopy. Our analyses highlight kinetic advantages for EAdVs in duodenal HuTu80 cell infection and reveal a larger fraction of mobile particles, faster virus uptake, and infectious particle entry in intestinal cells. Moreover, EAdVs display a dependence on clathrin- and dynamin-dependent pathways in intestinal cells. Detailed knowledge of virus entry routes and host factor requirements is essential to understanding pathogenesis and developing new countermeasures. Hence, this study provides novel insights into the entry mechanisms of a medically important virus with emerging tropism in a cell line originating from a relevant tissue.

**IMPORTANCE:**

Enteric adenoviruses have historically been difficult to grow in cell culture, which has resulted in lack of knowledge of host factors and pathways required for infection of these medically relevant viruses. Previous studies in non-intestinal cell lines showed slow infection kinetics and generated comparatively low virus yields compared to other adenovirus types. We suggest duodenum-derived HuTu80 cells as a superior cell line for studies to complement efforts using complex intestinal tissue models. We show that viral host cell factors required for virus entry differ between cell lines from distinct origins and demonstrate the importance of clathrin-mediated endocytosis.

## INTRODUCTION

Human enteric adenovirus types F40 and F41 (EAdVs) are the only human members of species F of the growing *Adenoviridae* family, which currently has over 110 members divided in species A–G (1, 2). While other adenovirus types cause disease in eyes, airways, liver, adenoids, and/or urinary tract, EAdVs have a pronounced tropism for the gastrointestinal tract (3) and are one of the leading causes for acute virus gastroenteritis and associated death in children below 5 years of age (4, 5). In 2022, more than 1,000 cases of acute hepatitis of unknown cause occurred in children worldwide, including 22 deaths (6). In the UK, adenoviruses were detected in 122 of 183 cases (69%), with human adenovirus type 41 (HAdV-F41) being the most common (92.3%) adenovirus type detected in blood (7). The exact role of EAdVs in acute hepatitis is still unclear, but it has been proposed that EAdVs facilitate replication of adeno-associated virus type 2 that is found transplanted livers at high viral load (8 – 10). This suggests a potential novel role for these viruses in human disease.

Despite being a common cause of disease in humans, the interplay of host factors and virus entry requirements for EAdVs is largely unknown. The main reason for this is that EAdV has been difficult to isolate and amplify to high viral titer in cell culture (11, 12). The icosahedral capsid of adenoviruses consists of three capsid proteins, where the major capsid protein, hexon, is the main structural component. Pentamers of viral penton proteins are located at the tips of the icosahedral vertices, and from their centers, trimeric fiber proteins are protruding (13). In contrast to other AdVs, EAdVs carry two distinct fiber proteins of different length (14, 15), where the short fiber mediates interaction with surface attachment factors and the long with receptors. The former are heparan sulfate proteoglycans (HSPGs), and among the latter is coxsackie and adenovirus receptor (CAR) (16, 17). All other AdV types interact with cell-surface integrins via an RGD motif in the penton protein, which aids in endocytic uptake and uncoating of the virus (18, 19). EAdVs, however, lack such conserved RGD motifs and, therefore, interact with laminin-binding integrins instead (20). The overall EAdV structure is largely resistant to pH changes that resemble passage through the stomach, which may contribute to the gastrointestinal tropism (21, 22). Due to the viruses’ fastidious nature, most studies have been conducted in A549 and HEK293 cells, where EAdV entry was slow, occurred in a clathrin-independent manner, and with inefficient endosomal escape (23, 24). Previous attempts to study EAdV infection in human ileal organoids succeeded in virus amplification but failed to identify EAdV target cells (25). This highlights the need to identify the molecular determinants responsible for the specific EAdV tissue tropism by exploring novel infection models.

In this study, we compared EAdV infection kinetics and cell entry pathway in duodenal HuTu80 cell lines with lung-derived A549 cell lines to identify possible cell type-specific advantages in cells derived from the gastrointestinal tract. We found that infectious internalization was faster and clathrin dependent in HuTu80 cells, highlighting the need for tissue-specific virus infection studies. Our results suggest that the clinically relevant but understudied EAdVs carry traits not seen in other adenoviruses, which enable efficient entry in cells of the gastrointestinal tract.

## RESULTS

### Faster uptake and infection kinetics of EAdVs in duodenal HuTu80 cells

**Fig 1:**
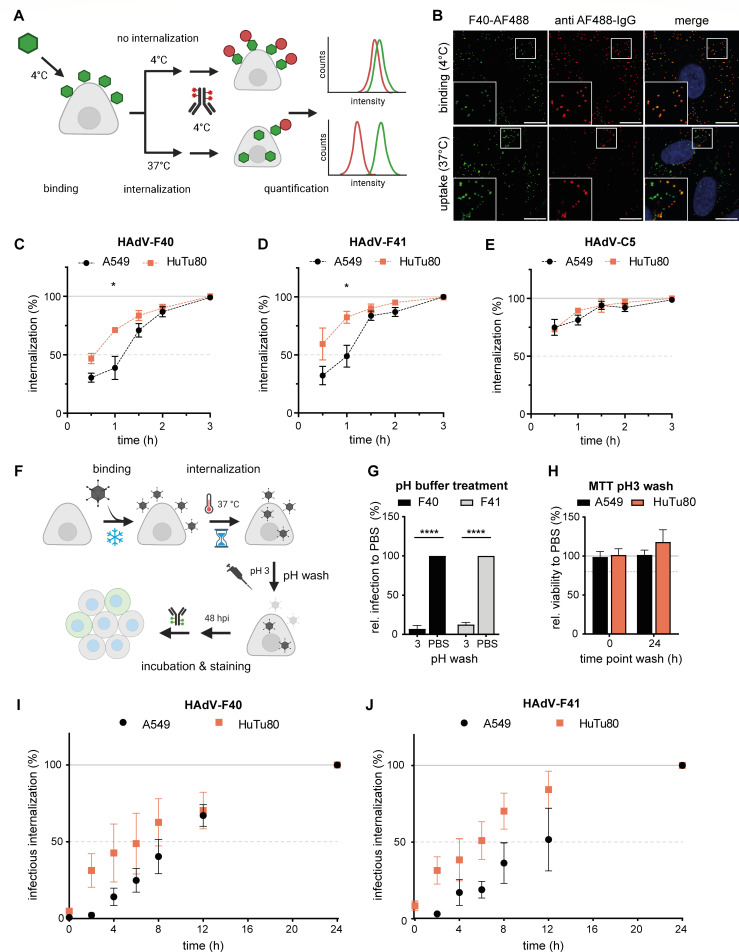
Uptake and infection kinetics of EAdV is faster in duodenal HuTu80 cells. (**A**) Scheme of the dual-label uptake assay to distinguish internalized from extracellular AdVs. AF488-AdVs were bound to target cells in suspension followed by internalization. Extracellular virus accessible to anti-AF488 antibody staining showed a dual labeling measurable by flow cytometry. (**B**) Validation of uptake of AF488-labeled HAdV-F40 bound to A549. Dual staining depended on extracellular localization as in the top panel with the binding only control as compared to 4 h uptake samples. Scale bar, 10 µm. (**C–E**) Uptake assay of HAdV-F40 (**C**), HAdV-F41 (**D**), and HAdV-C5 (**E**) in A549 (black) and HuTu80 (red) cells assessed through shifts in mean fluorescence intensities by flow cytometry. (**F**) Scheme of an infectious internalization assay to determine kinetics of infectious uptake of AdVs. (**G**) Sensitivity of EAdV infection to low pH wash after binding to A549 cells as detected by hexon-positive cells using automated microscopy. Error bars indicate the standard error of the mean. Statistical significance determined using two-way analysis of variance (ANOVA) test: ****P < 0.0001. (**H**) Low pH washes did not affect cell viability as tested by MTT assay at 48 h after pH 3 wash compared to phosphate buffered saline (PBS) wash at time points 0 h and 24 h. (I and J) HAdV-F40/F41 was bound to and internalized into A549 or HuTu80 cells. Extracellular EAdV particles were washed at low pH, whereby only internalized particles led to infection. Infection was assessed by immunofluorescence staining of hexon protein within cells. Error bars show standard error of the mean. Statistical significance by unpaired students *t*-test: *P < 0.05.

With the aim to explore host cell requirements of EAdVs in duodenal HuTu80 and lung-derived A549 cells, we established an uptake assay based on dual-labeling of virus particles (Fig. 1A). In brief, AF488 fluorescently labeled AdVs were bound to cells at 4°C followed by a shift to 37°C to allow internalization. To distinguish intracellular and extracellular virus particles, cells were shifted back to 4°C to inhibit further internalization, and extracellular particles were visualized with an anti-AF488 primary antibody and AF568 secondary antibody without fixation. Thereafter, live cells were analyzed for virus uptake by flow cytometry (Fig. 1C through E) for total virus signal (AF488) and extracellular fraction (AF568). To validate assay specificity, fluorescently labeled HAdV-F40 was bound to and incubated with cells at 4°C or allowed to directly internalize for 4 h at 37°C before staining on live cells with subsequent fixation (Fig. 1B). Analysis by confocal microscopy revealed that extracellular particles were double stained and dominant in the binding only sample (Fig. 1B, top row), whereas internalized particles were protected from anti-AF488 staining and only present in the uptake sample (Fig. 1B, bottom row). Of note here are the green only particles, which localized toward the perinuclear area, while dual-stained particles were localized to the cell periphery. EAdV was previously described to internalize slowly in A549 and HEK293 cells as compared to other AdV types (23, 24). We confirmed by flow cytometry that uptake of EAdV into A549 cells was slow and reached 90%–100% after 2–3 h, whereas 75% of HAdV-C5 particles were already taken up after 30 min (Fig. 1C through E). Interestingly, we observed that both HAdV-F40 and HAdV-F41 entered HuTu80 cells about twice as fast as A549 cells. In contrast, HAdV-C5 entered HuTu80 and A549 cells equally fast (Fig. 1E). This suggested a cell line-specific advantage for EAdVs in HuTu80 cells regarding virion internalization.

To investigate the relevance of this advantage for productive virus uptake, we next developed an infectious uptake assay, where EAdVs were bound at 4°C, then allowed to enter cells at 37°C for different durations. To inactivate virus particles after the desired internalization time, we tested a washing protocol with buffers in a pH range from pH 3 to pH 11. To our surprise, EAdVs were sensitive to washes with low pH (pH 3) and very high pH (pH 11) after cell binding and only resulted in low infection as compared to phosphate buffered saline (PBS) washes (Fig. 1G and data not shown). For the infectious internalization, we decided to use low pH washes as those have been used in other studies (26) and confirmed that pH 3 washes were not cytotoxic (Fig. 1H). After the respective internalization time, we rendered extracellular particles non-infectious by a brief low pH wash and quantified successful viral hexon production as a correlate of productive entry 48 h post infection. Both HAdV-F40 and HAdV-F41 reached higher infectious internalization levels in HuTu80 cells as compared to A549 cells, especially after washes at early time points between 2 h and 6 h of internalization (Fig. 1I and J). Thus, infectious EAdV internalization was faster in HuTu80 cells as compared to A549 cells. These results suggest that HuTu80 cells are a more suitable model to study EAdV entry and infection than previously used cell lines.

### Kinetic advantage is reflected in intracellular localization of EAdVs

**Fig 2:**
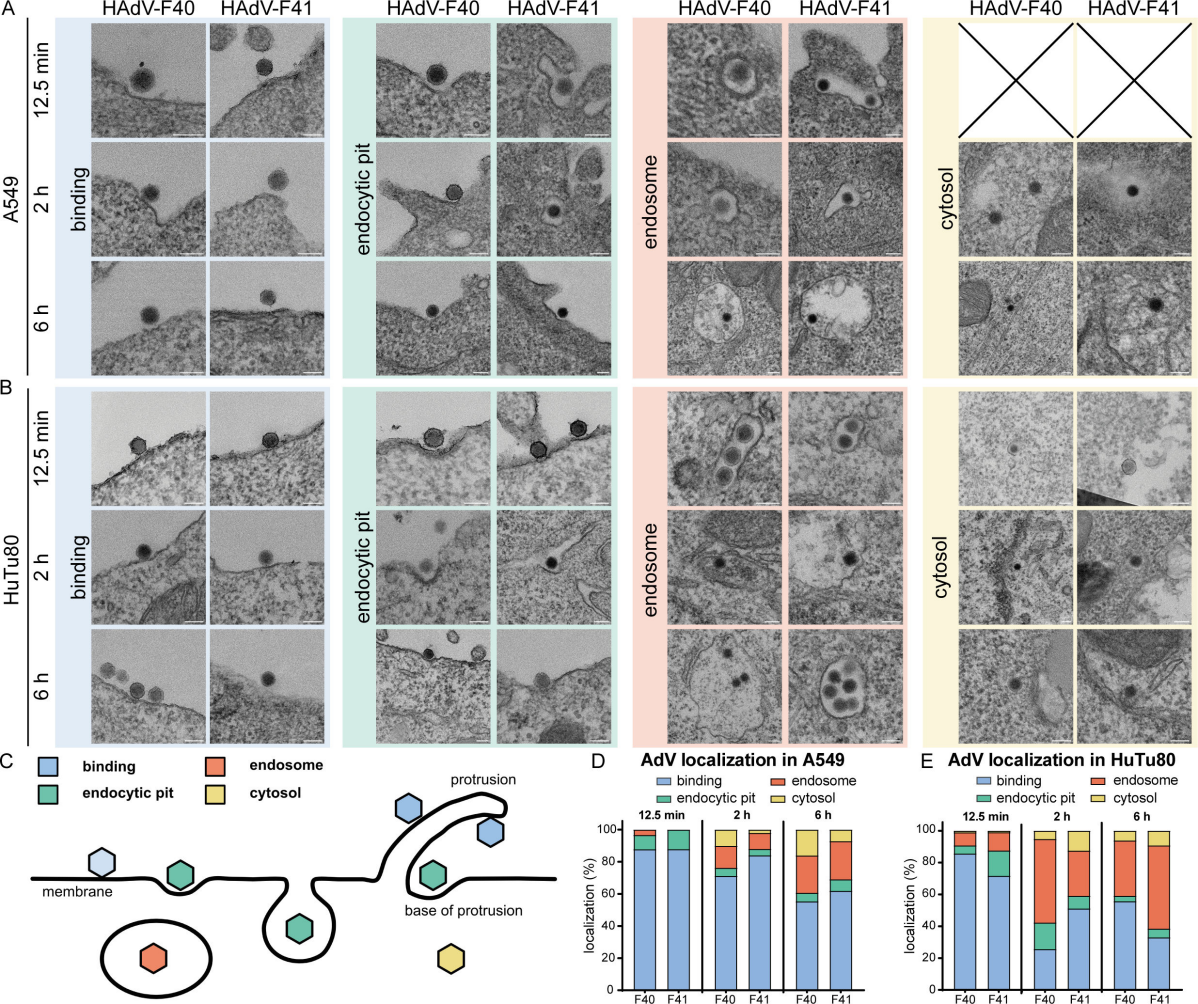
EAdV uptake observed in transmission electron microscopy mirrors kinetic advantage. (**A**) Panel of exemplary cropped TEM images from A549 cells incubated with HAdV-F40 (left) or HAdV-F41 (right) and classification of virus localization into categories: bound (blue), endocytic pit (green), internalized (red), and cytosolic (yellow). (**B**) Panel of exemplary TEM images as in (A) from HuTu80 cells. Scale bars: 100 nm. (**C**) Scheme for scoring of localization of AdV particles on cells in the EM experiments; (D/E) Quantification of uptake of EAdVs (F40/F41) into A549 (**D**) and HuTu80 (**E**) cells. At least *n* = 50 virus particles were classified per time point.

The observation that EAdVs entered HuTu80 cells rapidly prompted us to investigate EAdV localization in HuTu80 (Fig. 2B) versus A549 (Fig. 2A) after 12.5 min, 2 h, and 6 h of internalization by transmission electron microscopy (TEM). We consistently observed virus particles binding to flat membrane regions or at the tip of cellular protrusions (Fig. 2A and B, “binding,” and Fig. 2C, blue) at all time points and for both cell lines. Moreover, virus particles associated with curved membranes with and without clathrin coat were observed in all experimental conditions (Fig. 2A and B, “endocytic pit,” and Fig. 2C, green); virus particles were also found in potentially intracellular vesicles or endosomes (Fig. 2A and B, “endosome,” and Fig. 2C, red). In rare cases, virus particles appeared without endosomal membrane coat in the cytosol, indicating successful endosomal escape. However, these events were not observed in A549 cells at 12.5 min of internalization (Fig. 2A and B, “cytosolic,” and Fig. 2C, yellow). Quantification of these four virus localizations (plasma membrane, endocytic pit, endosome, and cytosol) indicated that at these early time points a greater fraction of viruses localized to intracellular compartments of HuTu80 as compared to A549 cells. This trend was most obvious at 2 h internalization, where only 15%–25% of HAdV-F40 and HAdV-F41 were found in big endosomal structures and the cytosol in A549 cells, but 40%–60% in HuTu80 cells (Fig. 2D vs Fig. 2E at 2 h). These results are well in line with our observations in flow cytometry and infection assays (Fig. 1). In summary, these data support that HuTu80 cells provide a cell type-specific advantage for EAdV infectious entry and may serve as an improved cell culture model.

### EAdVs display heterogeneous motion and increased free diffusion with time and in HuTu80

**Fig 3:**
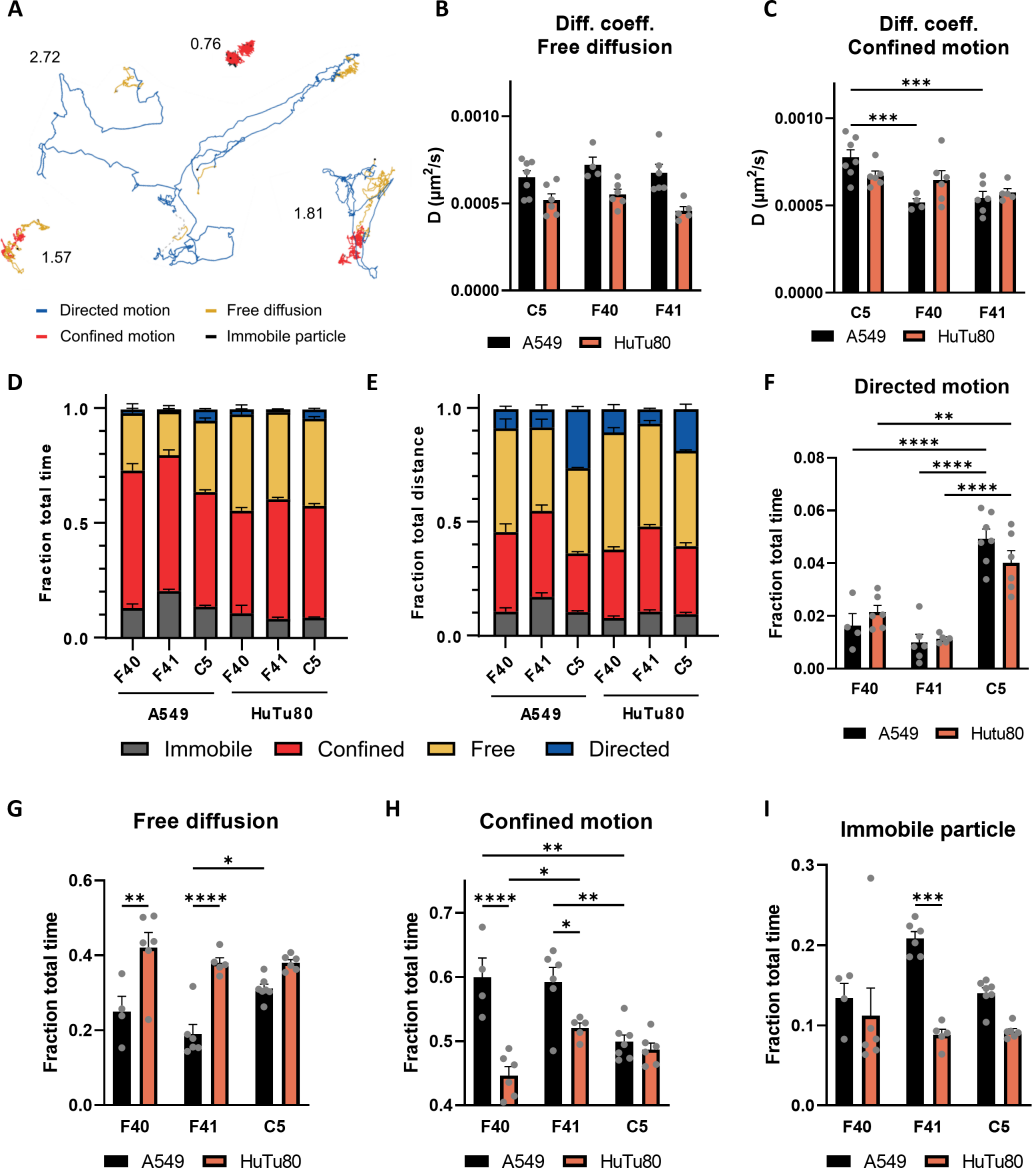
EAdVs display increased free diffusion on HuTu80 cells. (**A**) Representative tracks produced by HAdV particles moving on live cells. The colors show the results of the track segmentation algorithm. The number next to each track is its average motion coefficient as established in Fig. 4A. (B and C) Mean diffusion coefficient of HAdV particles displaying free (**B**) and confined (**C**) motion on A549 and HuTu80. (**D**) Fraction of the total track time spent by HAdV particles in each motion type on A549 and HuTu80. (**E**) Fraction of the total distance covered by HAdV particles in each motion type. (**F**) Fraction of the total track time spent by HAdV particles in directed (**F**), free (**G**), and confined (**H**) diffusion and immobile particles (**I**), on A549 and HuTu80. Gray dots indicate the value for each recorded video. All data were collected 30 min after the removal of the inoculum. Error bars indicate the standard error of the mean. Statistical significance determined using two-way ANOVA test: *P < 0.05, **P < 0.01, ***P < 0.001, ****P < 0.0001. Comparisons are performed between HAdVs on the same cell type and on the same HAdV on difference cell types.

After establishing that EAdV entry is significantly faster in HuTu80 cells than in A549, we investigated if virus particle motion after attachment to the cell surface also differed between cell lines. To this end, we tracked single particles of fluorescently labeled HAdV by highly inclined and laminated optical sheet (HILO) microscopy (27). We incubated A549 and HuTu80 cells with AF488-HAdV-C5/F40/F41 for 5 min at 37°C and observed virus motion on cells at 5 min and 30 min after removal of virus inoculum. We acquired videos at 10 frames/s for 2 min each. The tracks were analyzed using a divide-and-conquer moment scaling spectrum (DC-MSS) algorithm (28, 29), which allowed us to subdivide each track into segments displaying distinct motion patterns. Four motion types were considered in the analysis: immobile particles, confined diffusion, free diffusion, and directed motion. Representative examples of the acquired tracks are shown in Fig. 3A. We first analyzed the diffusion coefficient for each motion type. Surprisingly, no clear differences between HAdV types or cell lines were observed, except for a faster confined motion of HAdV-C5 on A549 when compared to EAdVs (Fig. 3B and C). Both free and confined diffusion had a mean diffusion coefficient between 0.0005 and 0.001 µm^2^/s, while directed motion exhibits slightly higher values between 0.0015 and 0.0035 µm^2^/s (data not shown). These values were comparable to values reported previously for HAdV-C2 internalization (19). In all cases and for all motion types, we observed large variations in the diffusion coefficient values, with standard deviations >0.02 µm/s and single recorded values ranging between 5 × 10^−5^ to 0.05 µm^2^/s. This heterogeneity in the diffusion coefficient value can be attributed to the different possible interactions between cell components and the virus, which result in various levels of mobility.

**Fig 4:**
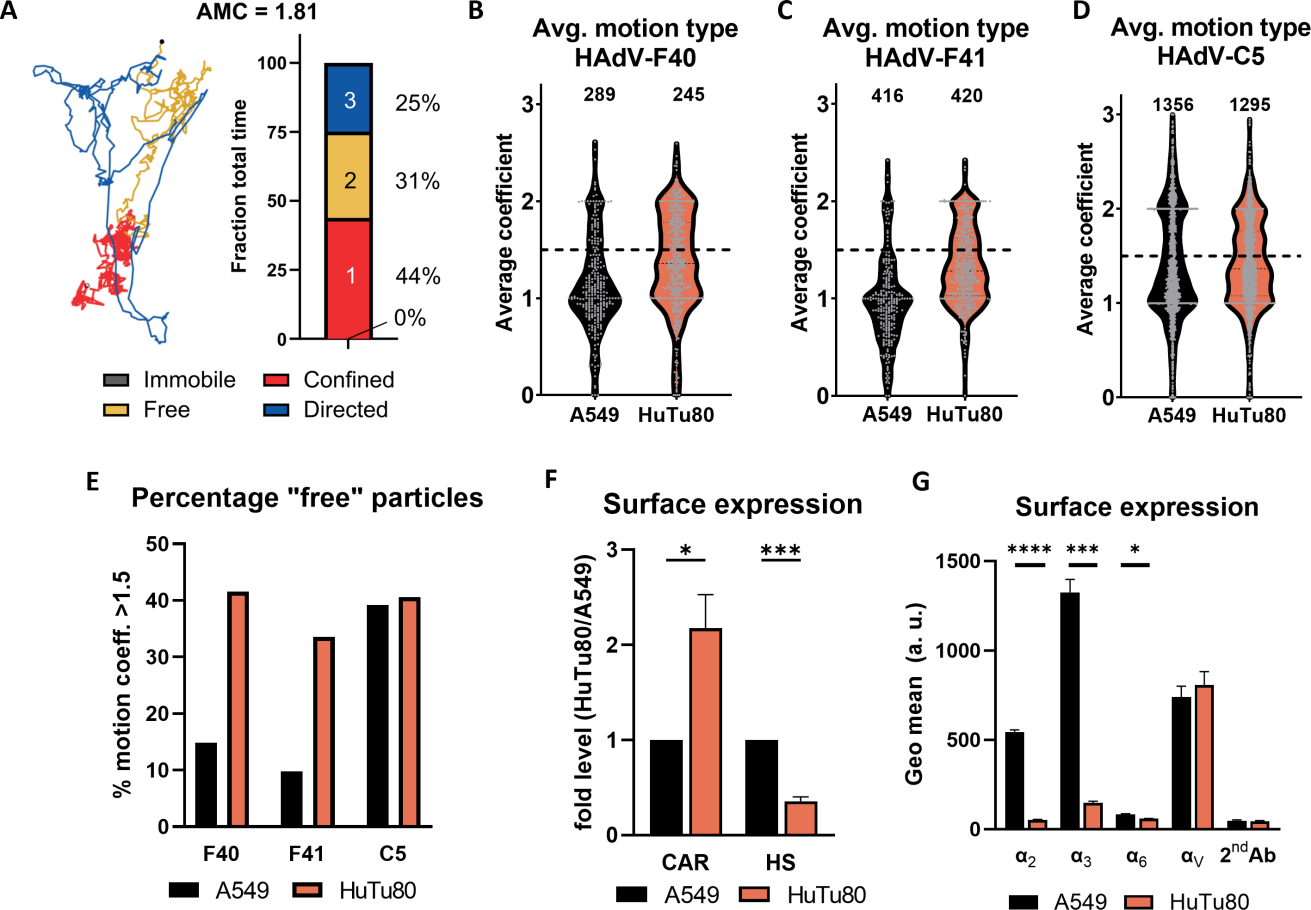
EAdV shows a larger mobile population on HuTu80 cells. (**A**) Example calculation of the AMC on a single track. The fraction of time spend in each motion type is multiplied by a coefficient: 0, for a fully immobile track; 1, for confined motion; 2, for free diffusion; and 3, for directed motion. (**B–D**) Truncated violin plot showing the distribution of full tracks according to their average motion coefficient on A549 and HuTu80 cells after 30 min from the removal of the inoculum for HAdV-F40 (**B**), HAdV-F41 (**C**), and HAdV-C5 (**D**). Single tracks are shown as gray dots. The number of tracks analyzed for each condition is displayed above each plot. (**E**) Percentage of the tracks with an average motion coefficient higher than 1.5, i.e., displaying a motion dominated by free and directed diffusion. (**F**) HuTu80 cells carry more CAR and less heparan sulfate (HS) than A549 cells as shown as the fold difference in surface expression level on HuTu80 over A549 cells. Errors displayed as standard error of the mean of *n* = 5 independent experiments. (**G**) Cell surface levels of selected α integrins involved in HAdV binding on A549 and HuTu80 cells. Displayed are geometric means of fluorescence signal from integrin-specific antibody staining and secondary antibody only control (2nd Ab) measured by flow cytometry. Errors displayed as standard deviation of *n* = 3 independent experiments. Statistical significance (F/G) determined using unpaired *t*-test: *P < 0.05, **P < 0.01, ***P < 0.001.

We then focused on the relevance of each motion type, measured as the fraction of time spent in each of those by the particles (Fig. 3D). We observed in all cases a prevalence of confined motion (from 40% to 68% of the total time) followed by free diffusion and immobile particles, with directed motion only relegated to a few percentage of the total time tracked. Noteworthy, the analysis of the fraction of total time likely underestimates the importance of fast but short-lived motion segments. In these segments, HAdV particles might cover large distances making them significant in the overall dynamic of the interaction with the host cell. To further explore this possibility, we considered the distance traveled by the particle in each segment as the distance between the two furthest points in each track segment. In this case, free diffusion becomes the most prevalent motion type, and the importance of directed motion becomes evident, accounting for as much as 20% of the distance travelled by HAdV-C5 particles on A549 cells (Fig. 3E). We then compared the variations in the frequency of each motion type between cell lines for all three viruses (Fig. 3F). The behavior of HAdV-C5 shows a larger fraction of directed motion when compared to EAdVs and little dependence on cell type (Fig. 3F). This supports the internalization data in Fig. 1 indicating a fast kinetics for HAdV-C5 internalization regardless of the cell type. Particularly noteworthy is the behavior of HAdV-F40 and HAdV-F41. As shown in Fig. 3G, when comparing motion on A549 and HuTu80 30 min after the removal of the inoculum, we observe a significantly larger fraction of particles undergoing free diffusion (0.25 to 0.42 and 0.19 to 0.38 for HAdV-F40 and HAdV-F41, respectively) and smaller fraction of confined motion (0.60 to 0.45 and 0.59 to 0.52 for HAdV-F40 and HAdV-F41, respectively) for HuTu80 cells. For HAdV-F41, we also observe a clearly lower number of immobile particles, while this is not the case for HAdV-F40 (Fig. 3I). This indicates a much more dynamic behavior of EAdV particles on intestinal cells. A similar behavior was observed 5 min after the removal of the inoculum (data not shown). Directly comparing the three viruses tested (Fig. 3F through I), we find that HAdV-F40 and F41 display a very similar behavior with small and mostly non-significant differences in all conditions, albeit showing a slight trend toward higher mobility for HAdV-F40 over F41. HAdV-C5, on the other hand, shows consistently higher mobility in both cell lines considered and in particular on A549 cells. In sum, we show that EAdVs display a similar and overall increased movement dynamics during the attachment and entry process in intestinal cells. Combining all segments of a motion type from all tracks, however, does not consider the possible heterogeneity in the diffusion behavior and mobility between single particles. To investigate this, we implemented a parameter that describes the overall dominant motion type for each track. We scored every motion type as 0 for immobile particles, 1 for confined motion, 2 for free diffusion, and 3 for free motion and defined the “average motion coefficient” (AMC) as the time average of the motion types displayed by each track. In this way, tracks receive a fractional number between 0 and 3, with values close to 1 indicating a predominantly immobile/confined motion and around 2 for free diffusion and directed motion. An example of calculation of the AMC score is shown in Fig. 4A, while the distribution of the AMC for all tracks 30 min after the removal of the inoculum is shown in Fig. 4B. In all conditions, the distribution of AMC values presents a peak at around 1, indicating a large percentage of particles display a predominantly confined motion. More interesting is the second peak present at AMC values of around 2. This population appears to be very small on A549 for EAdVs, while it is much larger and comparable to the confined peak on HuTu80 (Fig. 4B and C). As before, HAdV-C5 displays high mobility with a large population at AMC ~2 and a similar distribution in both A549 and HuTu80 cells (Fig. 4D). A similar behavior is observed 5 min post removal of the inoculum; however, in this case, the mobile fraction is significantly smaller than that at AMC ~1 suggesting overall fewer mobile particles at early time points (data not shown). The presence of two distinct populations indicates that the increase in free diffusion observed on HuTu80 (Fig. 3G) is due to the appearance of a population of mostly freely diffusing particles, while others remain immobile or restricted in their motion, rather than a homogeneous increase of the free diffusion mode shared by most particles. Finally, we estimated the size of the two populations by a simple thresholding at an AMC score of 1.5. After 30 min, the number of tracks undergoing mostly free diffusion is 2.8 times higher for HuTu80 than for A549 in the case of HAdV-F40 and 3.4 times higher for HAdV-F41. No clear increase is observed for HAdV-C5, when comparing the two cell lines (Fig. 4E). Taken together, we observe faster internalization of EAdV into HuTu80 cells that correlates with an increased overall mobility.

Virus surface movement is connected to their plasma membrane and receptor interactions (30). HAdV type C and F long fiber knobs bind CAR, while HAdV type F short fiber knobs interact with heparan sulfates (HS) (16, 17, 31). We investigated the EAdV virus preparations for short vs long fiber ratios and found that they contain more short than long fiber (data not shown) in line with published data (32). The affinities of HAdV-F41 and HAdV-C5 fiber knobs to CAR have been compared previously and shown to be similar with 7.3 ± 2.6 nM (F41) and 7.9 ± 1.8 nM (C5) (33). We compared affinities of immobilized HAdV-C5, HAdV-F40, and HAdV-F41 long fiber knobs to soluble CAR by surface plasmon resonance (Table 1). We found that affinities of F41 and C5 long fiber knob were similar with 6.34 ± 2.35 nM and 3.59 ± 1.49 nM, respectively. Interestingly, F40 long fiber knobs bound with weaker affinity to CAR: 25.47 ± 7.86 nM. This finding is unexpected as the reported protein sequence homology of type F HAdV fiber knobs is 97.7% (34) and, therefore, warrants future investigations. Nevertheless, as fewer long fibers are incorporated in HAdV-F40/F41 than in HAdV-C5 and the surface mobility profile of F40 and F41 was very similar, we conclude that long fiber:CAR interactions may—if at all—only have a minor influence on the cell type-specific differences observed between species F and C HAdVs.

**TABLE 1:** Summary of affinity assessment of HAdV fiber knob proteins and soluble CAR by surface plasmon resonance^*a*^

Serotype	*k*_d_ (s^−1^)	*k*_a_ (M^−1^ s^−1^)	*K*_D_ (M)
HAdV-F40	3.64 × 10^4^ ± 1.90 × 10^4^	7.86 × 10^−4^ ± 1.05 × 10^−4^	25.47 × 10^−9^ ± 7.86 × 10^−9^
HAdV-F41	2.80 × 10^5^ ± 0.91 × 10^5^	1.59 × 10^−3^ ± 4.37 × 10^−4^	6.34 × 10^−9^ ± 2.35 × 10^−9^
HAdV-C5	1.79 × 10^5^ ± 3.39 × 10^4^	5.97 × 10^−4^ ± 1.44 × 10^−4^	3.59 × 10^−9^ ± 1.49 × 10^−9^

^*a*^ Displayed are association (k_a_) and dissociation (k_d_) rate constants as well as the resulting affinity (K_D_) with standard deviation from three independent experiments.

To then explore fiber:receptor interactions as a possible cause of the observed differences in virus particle mobility, we assessed cell surface expression levels of AdV receptors. Our experiments revealed that HuTu80 carried about twice as much CAR but only a third of HS amount of A549 cells (Fig. 4F). This may hint at reduced HS trapping of EAdV particles on HuTu80 cells, explaining the larger population of mobile particles. We, furthermore, looked at surface expression of integrins, which have been previously described as receptors for viral penton base proteins (20). Here, EAdV differs from other HAdVs in that they are lacking an RGD motif (23), which mediates interaction of C-type HAdVs with RGD-binding integrins, i.e., αV containing heterodimers (18, 35). We checked the differences in surface expression level of integrins α3 and α6 (laminin-binding), αV (RGD-binding), and α2 (collagen binding) (36) on A549 and HuTu80 cells (Fig. 4G). We found that cell surface expression levels of α2, α3, and α6 were below detection limit in HuTu80, while αV showed a comparable cell surface expression level in A549 and HuTu80 cells. Taken together, we conclude that advantages in cell surface movement on HuTu80 cells may not be caused by HAdV-interacting integrins or CAR.

### EAdVs enter cells in a clathrin- and actin-dependent manner

Enteric adenovirus entry was previously studied in A549 and HEK293 cells found to be clathrin-independent in HEK293 (23, 24). The faster EAdV-specific uptake kinetics in duodenal HuTu80 cells prompted us to study their cell entry pathways using a flow cytometry-based uptake assay (Fig. 1A) in presence of small molecule inhibitors of endocytic pathways. Cells were detached with PBS/EDTA and suspended in growth medium with small molecule inhibitors for 30 min pretreatment. Next, we added virus particles to cells pretreated with inhibitors for 1 h at 4°C. Thereafter, the virus inoculum was removed, and cells were resuspended in medium with inhibitor and shifted to 37°C for internalization for 4 h. We used an anti-AF488 antibody staining on live cells at 4°C as above, to detect remaining extracellular virions.

**Fig 5:**
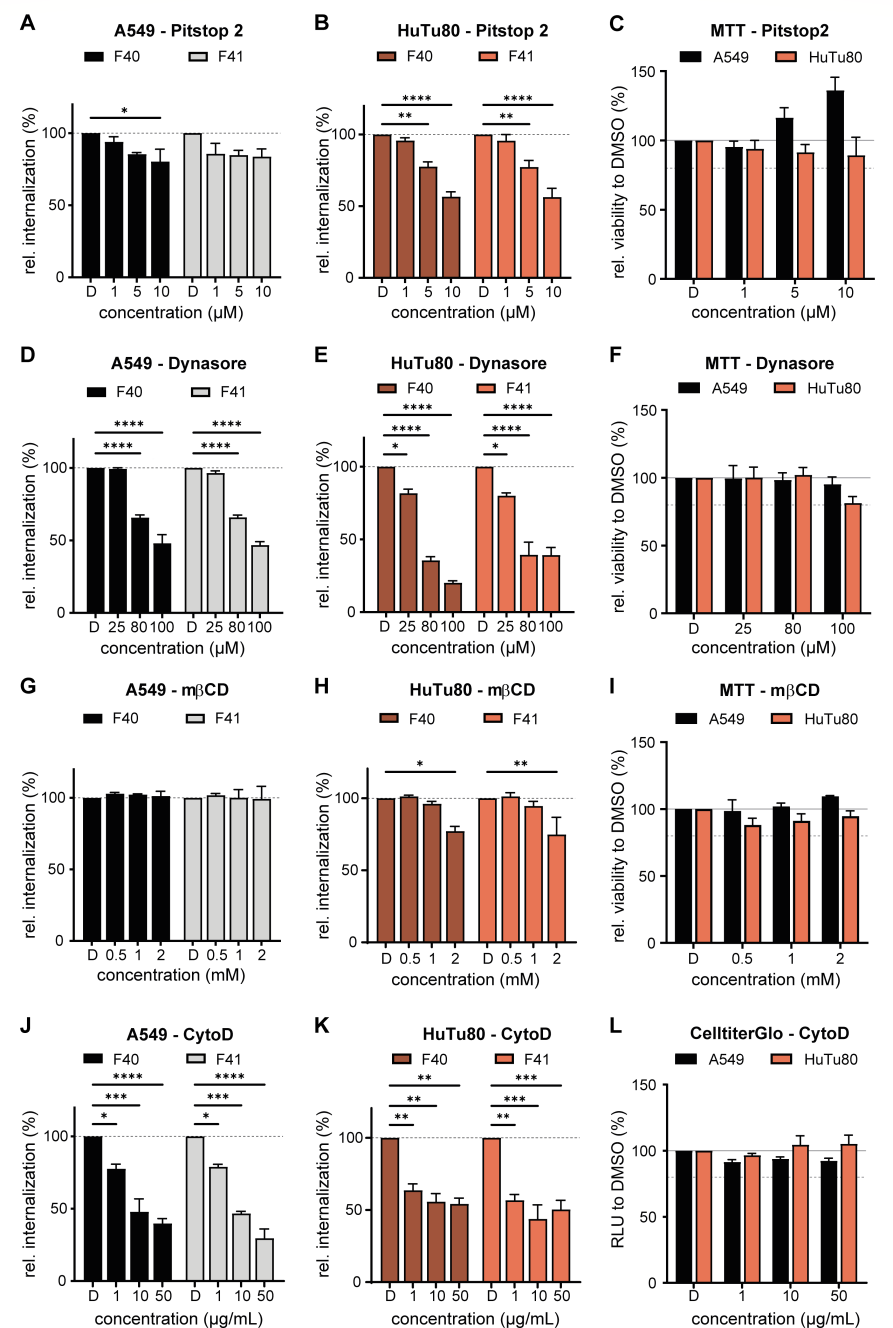
EAdV uptake depends on clathrin, dynamins, and actin. Relative EAdV uptake in A549 (black) and HuTu80 (red) cells in the presence of clathrin-inhibitor pitstop-2 (A and B), dynamin-inhibitor dynasore (D and E), cholesterol-depleting agent methyl-β-cyclodextrin (G and H), with actin-perturbating cytoD (J and K) at indicated concentrations, compared to vehicle control (DMSO). Error bars indicate the standard error of the mean. Statistical significance determined using two-way ANOVA test: *P < 0.05, **P < 0.01, ***P < 0.001, ****P < 0.0001. The absence of cytotoxicity of the 5 h inhibitor treatment was assessed by MTT assay for pitstop2 (**C**), dynasore (**F**), and methyl-β-cyclodextrin (**I**) and by CelltiterGlo assay for cytochalasin D (**L**).

Clathrin-mediated endocytosis (CME) is a well-characterized endocytic pathway (37) and used for cell entry by several AdVs (38). Pitstop2 blocks attachment of clathrin triskelions to the forming endocytic pit (39) and thereby inhibits CME. In our virus entry assay, pitstop2 reduced uptake of EAdVs dose dependently to a maximum of 50% internalization in HuTu80 cells but curiously did not affect uptake into A549 cells (Fig. 5A and B). We tested cell viability after pitstop treatment and did not detect any significant cytotoxicity (Fig. 5C). These findings prompted us to explore the involvement of CME further. To that end, we tested the involvement of dynamins, which are GTPases acting as important scission factors during endocytic vesicle formation in CME, but also during caveolae-dependent endocytosis and phagocytosis (40). We assessed the involvement of dynamins in EAdV endocytosis by inhibition of its GTPase function by dynasore (41). Dynasore inhibited EAdV uptake dose dependently in both cell lines. Specifically, dynasore reduced internalization rates to below 50% residual uptake for all viruses at the highest inhibitor concentration (Fig. 5D and E). At the same time, no cytotoxicity was observable at the same concentrations (Fig. 5F). As dynasore treatment affects several pathways, the larger reduction in internalization observed as compared to pitstop2 indicates that several endocytic pathways are active in parallel during uptake.

Next, we assessed whether EAdV entry required membrane cholesterol in addition to clathrin and dynamin. Cholesterol is an important membrane organizer and its depletion can affect membrane dynamics and domain formation (42, 43). We used methyl-β-cyclodextrin (mβCD) to sequester cholesterol from membranes (44, 45), which only mildly affected EAdVs uptake (Fig. 5G and H) and showed no cytotoxicity (Fig. 5I). This suggests that cholesterol-rich domains or cholesterol-dependent membrane dynamics are not involved in EAdV uptake.

Finally, we probed for the role of actin in EAdV entry. Actin dynamics affect, among other processes, the maintenance of membrane rigidity, cell movement, and intracellular transport (46). We utilized cytochalasin D (cytoD) to perturb the dynamics of the actin cytoskeleton by blocking actin filament elongation (47). Perturbation of dynamic actin remodeling by cytoD reduced EAdV uptake dose dependently to maximally 50% residual particle uptake in HuTu80 and 60%–70% in A549 cells (Fig. 5J and K). CytoD treatment induces efficient cell rounding due to the loss of the actin cytoskeleton; however, it did not induce cell death in this assay (Fig. 5L). Loss of virus uptake, however, was expected, as actin regulates several cellular processes and thus can act at several stages.

In summary, our data from an uptake assay suggest that EAdV entry is clathrin independent in A549 cells, but clathrin and dynamin dependent in HuTu80, pointing toward CME as the major uptake route in duodenal cells. Therefore, we decided to corroborate our findings on particle uptake by testing if infectious EAdV entry was clathrin dependent.

To ensure that the drugs are effectively blocking endocytosis at the concentrations used in A549 and HuTu80 cells, we performed infection experiments with vesicular stomatitis virus (VSV). VSV enters cells by clathrin-, dynamin-, cholesterol-, and actin-dependent endocytosis [summarized in reference (48)] and thus serves as positive control. In agreement with published data, we found that VSV infection was dose dependently blocked by the inhibitors used above (data not shown).

**Fig 6:**
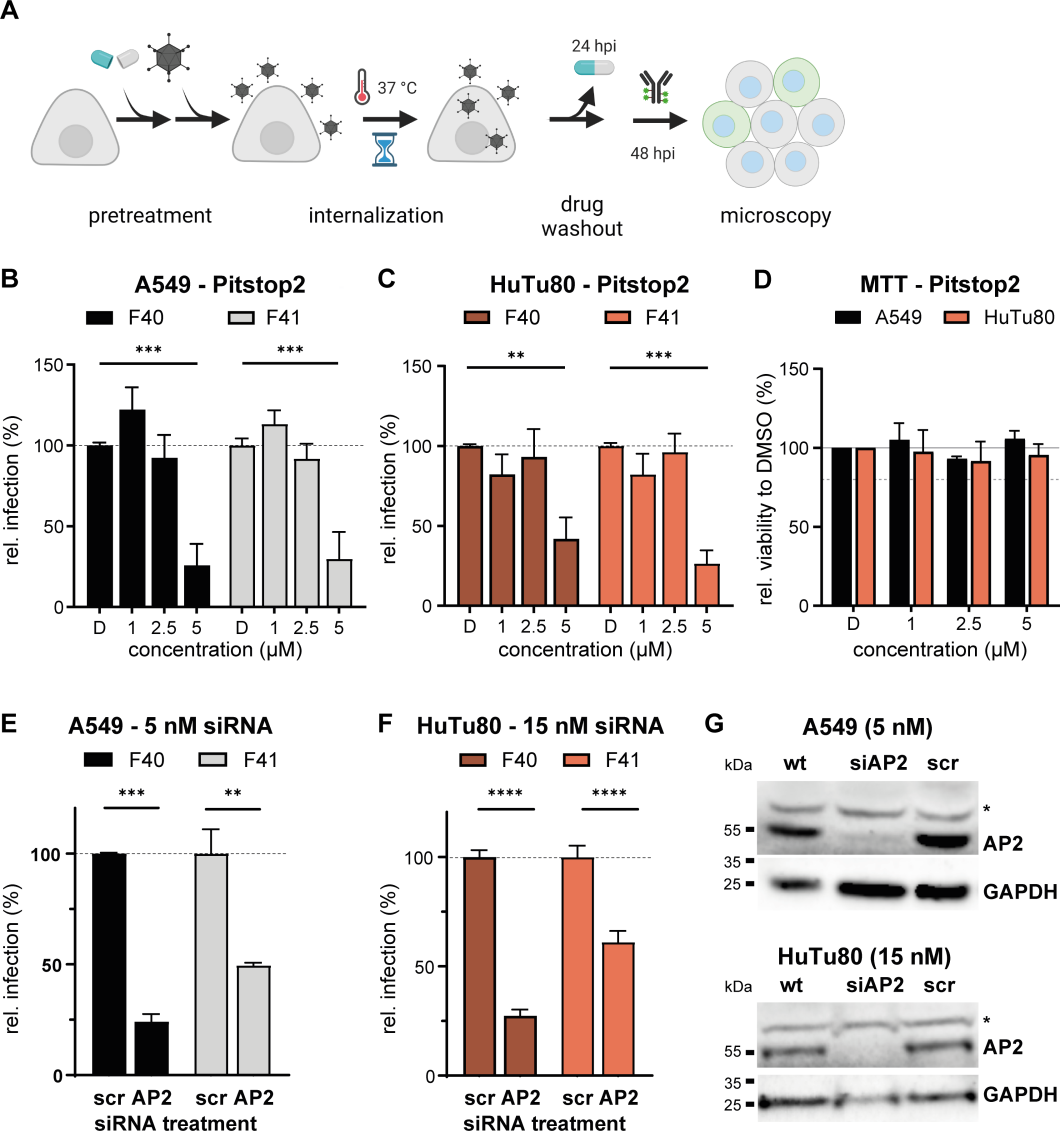
Infectious internalization of EAdV depends on clathrin in HuTu80 cells. (**A**) Scheme of the infectious internalization assay in presence of inhibitors. (**B and C**) Infectious internalization in the presence of pitstop2 in A549 (black) and HuTu80 (red) cells. (**D**) MTT assay showed low cytotoxicity of the drug treatment. (E and F) Infectious internalization after treatment of cells with siRNA against clathrin adapter AP2 or non-targeting siRNA scrambled control. (**G**) Representative Western blot after siRNA knockdown of AP2 in A549 (top) and HuTu80 cells (bottom). Efficient knock down was observed in both cell types. An unspecific band for the AP2 antibody is marked with asterisk (*). GAPDH served as loading control. Error bars indicate the standard error of the mean. Statistical significance determined using two-way ANOVA test: *P < 0.05, **P < 0.01, ***P < 0.001, ****P < 0.0001.

Consequently, we established an infection setup optimized for cell survival and stable EAdV hexon readout. Due to the slow infection life cycle of EAdV, stable hexon signal was not detectable before 44–48 h post infection. We treated HuTu80 and A549 cells with pitstop2 for 30 min before addition of HAdV-F40 or HAdV-F41. The cells were then incubated for 2 h at 37°C until the inoculum was exchanged for medium containing inhibitor. The inhibitor was removed at 24 h post infection to increase cell viability within the assay, and cells were fixed and stained for viral hexon and nuclei at 48 h post infection. Interestingly, pitstop2 reduced EAdV infection of HuTu80 and A549 cells only at the highest inhibitor concentration, but not in a dose-dependent manner (Fig. 6B and C). At 5 µM pitstop2, infection of both cell lines with HAdV-F40 and HAdV-F41 was reduced by more than 50% compared to the DMSO-treated control. Cell viability was not affected at these concentrations (Fig. 6D). From these data, we conclude that both overall particle uptake and infectious EAdV uptake in HuTu80 were clathrin dependent.

To verify our findings omitting pitstop2, we used RNA interference against AP2, a clathrin adapter at the plasma membrane (49), followed by infection with EAdV. To generate an efficient knock-down level, we depleted AP2 in HuTu80 and A549 cells by double transfection with 48 h in between with siRNA against AP2 or with a scrambled control (50). We seeded cells 24 h after the second siRNA treatment, infected them 24 h later as stated above, and fixed and stained for hexon protein and nuclei at 48 h post infection. We harvested additional cells at the time of infection to determine knock down efficiencies on protein level, which were optimized for each cell line (Fig. 6G). Knock down of AP2 reduced infection of HuTu80 by 75% for HAdV-F40 and 40% for HAdV-F41 and infection of A549 by 75% for HAdV-F40 and 50% for HAdV-F41 (Fig. 6E and F). Infection of A549 cells appears to be clathrin dependent despite an independence of EAdV overall uptake in presence of pitstop2 (Fig. 5A). This suggests additional clathrin-independent non-productive uptake pathways in A549 cells. In contrast, our uptake and infection assays for HuTu80 consistently suggest a role for CME in EAdV infection. Interestingly, HAdV-F41 infection was affected less by AP2 knock down in A549 and HuTu80 cells than HAdV-F40, which may hint at an additional entry pathway for HAdV-F41. Taken together, we conclude that EAdV can rapidly infect HuTu80 cells in a clathrin-dependent manner.

## DISCUSSION

In this study, we aimed to evaluate the infectious pathway of EAdVs in HuTu80 with intestinal background compared to lung-derived A549 cells. Our results show that EAdVs have a kinetic advantage when infecting HuTu80 over A549 cells. Furthermore, we find that EAdV enter cells via a clathrin-dependent mechanism in A549 and HuTu80 cells, while clathrin-independent EAdV uptake was described for HEK293 cells. A faster and possibly more efficient uptake in HuTu80 cells is well in line with EAdV’s natural tropism in the gastrointestinal tract. Differential host cell requirements in uptake pathways and their translational implications have recently become obvious. For example, SARS-CoV-2 enters VeroE6 cells by endocytosis but enters lung epithelial cells by fusion at the plasma membrane. Thus, chloroquine, an inhibitor of cathepsin L in endolysosomes, has antiviral effects against SARS-CoV-2 in Vero cells, but not in cells of lung tissue origin (51, 52). This underlines that infection models reflecting the target tissue are vitally important to understand mechanisms of virus entry and infection and to device strategies for antiviral treatment.

Historically, EAdV was fastidious and difficult to grow to high infectious titer in tissue cultures (11, 12). Even to date, we observe that overall yields and infectious-to-non-infectious particle ratios are poorer than for other adenoviruses (e.g., HAdV-C5). Despite their relevance as a leading cause of acute infantile gastroenteritis (4), EAdV remains understudied (53). EAdVs are found in stool samples from children with acute gastroenteritis, but we lack knowledge of their initial target cells or target regions in the gastrointestinal tract. Hence, virus entry studies originate from easily accessible cell lines like A549 and HEK293 cells. In these studies, EAdV uptake into A549 cells was slow, and a major fraction of particles were still neutralized by antibodies after 4 h (23). Similarly, HAdV-F41 entry into HEK293 was inefficient (24). These observations prompted us to study EAdV host factors in cells originating from the gastrointestinal tract. Our results show a faster uptake of EAdVs into HuTu80 cells when directly compared to A549 cells. As the gastrointestinal tract is specialized on nutrient resorption, it is tempting to speculate that the kinetic advantage lies in a different overall endocytic activity in HuTu80 cells. For this reason and as recycling of particles back to the cell surface cannot be excluded in uptake assays, it is critical to discriminate between productive and unproductive endocytic uptake of virus particles. To this end, we compared not only global virus uptake but also uptake resulting in successful production of capsid protein (i.e., infection). Consistently, we found a faster productive uptake of EAdVs in HuTu80 cells as compared to A549 cells but no enhanced uptake kinetics for HAdV-C5. This suggests that the infectious route of EAdVs rather than overall endocytic activity was enhanced in HuTu80 cells.

We hypothesized that the observed difference in internalization behavior between A549 and HuTu80 cells may result from cell type-dependent surface interactions and/or differential availability of surface receptors, which could, in turn, affect particle mobility at the cell surface. For this reason, we tracked particles on the cell surface and analyzed their trajectories. We performed single particle tracking of EAdVs and HAdV-C5 on HuTu80 and A549 at early time points after binding, when we suspected most particles to be on the cell surface based on our uptake and infectious internalization kinetics (Fig. 1). We found high variability in the measured diffusion coefficient for all conditions and motion types. We speculate that the variability arises from the multiple possible interactions that occur between AdVs and the cell surface. EAdVs interact with CAR via the knob domain of their long fiber (17) and with heparan sulfate via the knob domain of their short fiber (16). Moreover, HAdV-F41 penton base can engage laminin-binding integrins as co-receptors in human colon cancer cells, which no or few other adenovirus types do (20). The exact receptor interactions for successful cell entry on A549 cell or intestinal cells, however, remain unknown. Strong multivalent interactions between cellular receptors and the virion fiber proteins may cause immobile or slow diffusing confined particles, as it would be the case with particles in endocytic pits but also during interaction with attachment factors like HSPGs after initial binding. Higher mobility of particles can arise from binding to mobile receptors on the cell membrane, which may either display free or confined diffusion or be coupled with the underlying moving matrix. The latter coupling results in directed motion on the plasma membrane, as suggested for HAdV-C2 in human embryonic retinoblast 911 cells (19). Similarly, extracellular matrix coupled motion was observed for other viruses in an actin-dependent manner (30, 54, 55) or even in a microtubule-assisted manner during tracking of the C-type lectin DC-SIGN in dendritic cells (56). In addition, our method, although confining the observation to few hundreds of nanometers across the apical membrane of the cell, does not allow to distinguish between external and internalized particles. Thus, a small part of the observed motion is likely to be attributed to the movement of endosomes containing viral particles in the proximity of the membrane.

Observing the predominant motion types, we showed a significant increase of free diffusion and a respective reduction of confined particles in HuTu80 cells compared to A549, particularly 30 min after attachment (Fig. 3G through H). This can be, at least partially, explained by HuTu80 cells carrying less HSPGs but slightly more CAR on their cell surface than A549 cells (Fig. 4F); EAdV may be slowed by multivalent interactions with HSPGs on A549 and have a surface sampling advantage leading to faster internalization in HuTu80 cells. This hypothesis is also supported by the finding that large extracellular domains, like HSPGs, have smaller diffusion coefficients on giant luminal vesicles (57). To validate this hypothesis, virus particles with short or long fibers only would be valuable tools to assess the contribution of known receptors to the entry kinetics and diffusion behavior.

Finally, to study the motion of single virions, we analyzed the overall motion behavior of each particle by implementing an AMC. Here, we found that the increase in free motion on HuTu80 is due to a larger fraction of the particle population showing a higher mobility, i.e., AMC score >1.5 (Fig. 4E). We speculate that the greater fraction of mobile particles reflects viruses bound to mobile receptor species possibly scouting the surface for their site of internalization. We, indeed, observe a correlation between the increase in mobility and faster entry kinetics. EAdVs show a larger mobile fraction in HuTu80 cells than in A549, corresponding to the faster internalization observed in the entry assay. This is also true for HAdV-C5, which is characterized by a particularly rapid entry in both cell line tested and which is consistently the virus showing the highest mobility. Furthermore, an increase in mobility has also been observed for HAdV-C2 interacting with its receptor CAR prior to endocytic uptake (19), indicating that faster receptor engagement may be an important factor in the improved entry kinetic observed in duodenal cells over A549.

EAdVs enter the intestine by passing through the stomach with a low pH milieu. Previous studies demonstrated that EAdV particles are resistant to low pH explaining in part their tropism (21). In our study, we established a low pH wash protocol to block EAdV entry into intestinal epithelial cells. While this seems counterintuitive, we could reproducibly show that cell-bound EAdV particles become non-infectious upon low pH wash in contrast to cell-free pH-treated EAdVs (21, 22). Our results indicate that EAdV-cell surface molecule interactions may induce a conformational change in the EAdV capsid that renders the virus particles acid sensitive. This is in line with a sequence of entry cues for EAdV as seen for HAdV-C2 in a step-wise process (19, 58), starting with attachment to host factors followed by secondary low pH priming in endosomes, which then triggers endosomal membrane rupture and escape. Future work will address whether such sequential entry cues govern EAdV entry into the intestinal epithelium.

Here, we report the first TEM imaging of EAdV particles entering cells of duodenal tissue origin. In TEM, EAdV increasingly localized intracellularly (endosomal structures and cytosol) in both cell lines over time reflecting successful virus entry and endosomal escape. The effect was again more pronounced in HuTu80 cells and strongest after 2 h of internalization. This was in line with the effects seen in the early phases (0–6 hpi) in the infectious internalization kinetics and supporting the kinetic advantage observed. Virus particles were mainly observed on smooth membranes and membrane invaginations lacking a visible clathrin coat structure indicating a non-clathrin-dependent entry pathway. We demonstrated, however, that productive virus uptake and infection were clathrin dependent in inhibition and RNAi assays. This can be explained by EAdV’s slow and protracted entry, where single virus entry events likely occur asynchronously and over an extended period of time as seen for other viruses, e.g., human papillomavirus 16 (26). In contrast, CME is a fast process, which occurs within 2 min (59). In our assays, we are, therefore, observing single internalization events at any time point rather than a synchronous entry of many particles. Those events themselves may be fast as they occur by CME. In static experiments like TEM, where we are observing cells at a fixed time point, we hypothesize that we likely miss most endocytic events. We observed a small fraction of viruses in endocytic pits mostly without a clathrin coat, which could indicate that those endocytic events are mechanistically slower or possibly delayed due to inefficient cargo recognition. Orthogonal methods including pharmacological perturbation and RNA interference are clearly better suited to reveal involvement of fast uptake mechanisms. Nonetheless, our TEM analysis confirms a faster uptake of EAdVs into cells of intestinal origin, and our data collectively argue for including intestinal epithelial cell lines in the toolbox of EAdV studies.

Our pharmacological perturbation and RNA interference studies reveal clathrin-mediated endocytosis as the major entry pathway of EAdVs into intestinal cells. This is in contrast to previous studies on HEK cells (24) and to our observations for uptake in A549 cells, highlighting the importance of choice of experimental system. During infectious internalization experiments, pitstop2 only showed a partial reduction of infection at the highest inhibitor concentration. Due to high cytotoxicity and possible off-target effects, the drug was washed out after half of the incubation time. This may be the reason for a lack of dose dependency for pitstop2, as successful entry and hexon production may be possible after wash-out of the drug. Because of these limitations and the debatable specificity of pitstop2 (60, 61), we additionally confirmed importance of CME for EAdV entry by RNA interference with the clathrin adapter AP2. Furthermore, we included actin perturbation by cytoD in our infection experiments using a similar setup. Cells showed a strong rounding because of the depolymerizing actin cytoskeleton, which leads to a partial cell loss during medium exchange after 24 h in infection and cytotoxicity assays. We observed a dose-dependent reduction of infection in presence of cytoD indicating actin-dependent endocytosis. However, as cytoD induced some cell death, we excluded the data from the study (data not shown). Taken together, through orthogonal assays, we demonstrated an important role of CME in productive EAdV entry into intestinal epithelial cells. As the cell system used originated from tissue of EAdV tropism, we propose that CME is a physiological uptake route. Studies using human intestinal organoids need to confirm this notion.

As both enteric adenoviruses were used in all our experiments, our study also allows for the comparison of the entry behavior between HAdV-F40 and HAdV-F41. They display a very similar behavior with no apparent differences in internalization kinetics, both in terms of global and productive uptake. A slight trend toward higher mobility for HAdV-F40 is observed in single-particle tracking experiments, with a longer time spent in free motion in both HuTu80 and A549. However, the differences are quite small and rarely significant, especially compared to the ones observed between EAdVs and HAdV-C5 and that between cell lines. Inhibition experiments also showed an almost identical drug response for the two EAdVs. HAdV-F40, however, seems to be more sensitive to siRNA inhibition of AP2, indicating that HAdV-F40 might depend to a larger extent on CME for internalization. Aside from small differences, HAdV-F40 and HAdV-F41 show a very similar entry behavior, with increased entry kinetics and higher mobility in duodenal cells and use CME as their main entry pathway.

Our study highlights the importance of studying host factors and infection pathways in appropriate model systems. In contrast to previously used respiratory epithelial cell models, EAdV infection of HuTu80 cells with duodenal background revealed that EAdV entry is clathrin dependent and that enhanced infection kinetics may be a measure for infection efficacy. Clearly, cell lines from different tissue backgrounds will remain an important tool to identify and validate host factors during virus infections. This study characterizes duodenal cancer cells as a novel tool for the study of EAdV and reveals previously neglected aspects of the cell entry mechanisms of these medically relevant viruses.

## MATERIALS AND METHODS

### Cells, media, and chemicals

A549 and HuTu80 cells cultured in Dulbeccos modified Eagle medium(DMEM, Sigma, #D5648) supplemented with 1× pen-strep (Gibco, #15140122) and 20 mM HEPES (Thermo Fisher Scientific, #BP310-500) (DPH) and 10% fetal bovine serum (FBS, Hyclone, #SV30160.03) at 37°C and 5% CO_2_. For infections, medium without FBS was used for incubation (DPH) and changed to medium with low FBS until fixation (DPH + 2% FBS). Small molecule inhibitors were pitstop2 (Abcam, #ab120687), dynasore (Abcam, #ab120192), cytochalasinD (Abcam, #ab143484), and methyl-β-cyclodextrin (Sigma, C4555-1G). CsCl was from Sigma (C4036).

### Viruses

HAdV-F40 (strain Hovix), HAdV-F41 (strain Tak), and HAdV-C5 (strain adenoid 75) were produced in A549 cells and purified as described previously (62). In brief, detached cells were pelleted at 800 rpm, supernatants were discarded, and cell pellets were suspended in DMEM. For cell lysis and virus extraction, cell suspensions were supplemented with an equal volume of Vertrel XF (Sigma; #94884) and shaken thoroughly. Phases were separated by centrifugation for 10 min at 3,000 rpm. The upper aqueous phase was loaded onto a CsCl step gradient, where the cushion with density 1.27 was underlayed with density 1.32 and density 1.37 CsCl solutions for ultracentrifugation in a SW40 rotor (Beckman Coulter) for 2.5 h at 4°C and 25,000 rpm. The virus containing band was extracted from the gradient and desalted on a NAP column (VWR, Cytiva, Illustra NAP) by elution in PBS, pH 7.4, and frozen at −80°C after addition of 10% glycerol.

### AF488-labeled AdV

AdV stocks were produced as above and used frozen (HAdV-C5) or fresh (HAdV-F40 and HAdV-F41) for covalent labeling with AF488-NHS (Thermo Fisher Scientific; #A20000). Viruses were used at a concentration of at least 500 ng/µL (best 1.000 ng/µL) and incubated with 10-fold molar excess dye over virus in a 500 µL reaction for 1 h rotating and light protected at room temperature. Virus was separated from free dye by ultracentrifugation as above on a SW60Ti rotor (Beckman Coulter). The virus band was extracted and desalted on a NAP column (VWR, Cytiva, Illustra NAP) by elution in PBS, pH 7.4, and frozen in small aliquots at −80°C after addition of 4% glycerol.

### Immunofluorescence staining

A total of 75,000 A549 cells were seeded in growth medium on ibidi 8-well µ-slides (ibidi, #80826) 24 h prior to experimentation. AF488-labeled AdVs were bound to cells at 4°C on ice for 1 h. Medium was exchanged, and cells were either kept at 4°C or shifted to 37°C for 4 h for internalization. Samples were transferred to 4°C for staining with anti-AF488-antibody (rabbit, Thermo Fisher Scientific; #A-11094) for 30 min on ice shaking, washed once with cold PBS + 2% FBS, and subsequently stained with anti-rabbit IgG-AF568 for 30 min at 4°C shaking. Cells were fixed with 4% paraformaldehyde (PFA)/PBS for 20 min and stained with AF647-Wheat Germ Agglutinin (WGA, Thermo Fisher Scientific, #W32466) and Hoechst33342 (Thermo Fisher Scientific; #62249). Samples were imaged as z-stacks with a 63× objective on a Leica SP8 confocal microscope at the Biochemical Imaging Center Umeå. Images were processed by maximum intensity projection with Fiji (63).

### Uptake assay

Cells were detached with PBS/EDTA, pelleted, suspended in DPH + 10% FBS, and kept in suspension on a shaker for 1 h for recovery. A total of 2 × 10^5^ cells per well were pelleted in a v-bottom 96-well plate and washed with cold DPH. For uptake assays in presence of inhibitors, cells were preincubated with inhibitor dilutions in DPH for 30 min before subsequent virus incubation. Cells were resuspended in 100 µL DPH (with or without inhibitors) containing AF488-labeled viruses (about 100 particles per cell) and incubated on ice for 1 h. Cells were pelleted and washed once with 100 µL cold DPH. Control samples were left on ice, and internalization samples were transferred to 37°C for 4 h. Cells were pelleted and incubated with anti-AF488-antibody (rabbit, Thermo Fisher Scientific; #A-11094) for 30 min on ice shaking, washed once with cold PBS + 2% FBS, and subsequently incubated with anti-rabbit IgG-AF568 for 30 min at 4°C shaking. Cells were washed once with cold PBS + 2% FBS and resuspended in 50 µL cold PBS + 2% FBS. Samples were measured by flow cytometry (BD Accuri) to assess mean fluorescence intensities of AF488 and AF568.

### Infectious entry assay

A total of 25,000 A549 or 30,000 HuTu80 cells per well were seeded in a black optical bottom 96-well plate (Greiner Bio-one; #655096) 24 h prior to experimentation.

#### Titration

Virus stocks were titrated for every batch to determine a dilution that results in 20% absolute infection. Virus stocks were diluted serially in DPH; 50 µL of the virus dilution was added to cells and incubated for 2 h at 37°C. Inoculum was removed; samples were supplemented with DPH + 2% FBS and incubated until 48 hpi. Cells were fixed by addition of 8% paraformaldehyde in PBS to the culture medium and incubated at room temperature for 15 min.

#### pH wash

pH sensitivity of EAdV infection after cell surface binding was tested by a 2 min pH wash after virus binding in DPH for 1 h on ice. Cells were incubated for 2 min with either 0.1 M citrate/PBS buffer (pH 3–6) or 0.1 M CAPS/PBS (pH 8–11) or PBS (pH 7.4). Samples were neutralized by two washes with PBS and subsequent addition of DPH + 2% FBS until fixation with 8% paraformaldehyde in PBS at 48 h post infection.

#### Kinetics

Cells were washed once with DPH without FBS and transferred to 4°C. Medium was removed, and 50 µL virus dilution was added to each well for binding at 4°C for 2 h. Inoculum was removed, and warm DPH + 2% FBS was added to each well. At each time point, duplicate samples were washed with 0.1 M citrate buffer pH 3 for 2 min, subsequently washed gently with PBS, and supplemented with warm DPH + 2% FBS until fixation with 4% paraformaldehyde in PBS at 48 h post infection.

#### Inhibitor

Cells were incubated with inhibitors dilutions in DPH for 30 min at 37°C. Meanwhile, virus dilutions were mixed with the inhibitor dilutions in a dilution plate. Medium was removed from the cells, and 50 µL virus-inhibitor dilution was added to each well for initial binding and internalization for 2 h at 37°C. Then, inoculum was removed, and 100 µL inhibitor dilutions mixed with DPH + 4% FBS were added to each well for 24 h. Thereafter, medium was removed, and 100 µL warm DPH + 2% FBS was added until fixation with 4% paraformaldehyde in PBS at 48 h post infection.

#### Staining

Cells were permeabilized with 100% ice-cold methanol for 20 min at −20°C and rehydrated by washing with PBS and then stained for viral hexon protein expression with an anti-hexon primary antibody (mouse; Merck; #MAB8052) and an anti-mouse secondary antibody coupled to AF488 or AF647 (Thermo Fisher Scientific; #A-11001/#A-21235). Nuclei were stained with Hoechst33342 (Thermo Fisher Scientific; #62249) and used to determine cell numbers. Plates were scanned in a Biotek Cytation5 automated microscope with a 10× objective with subsequent image analysis for cell count and infection counting or using the Tina program a TROPHOS plate reader (Luminy Biotech Enterprises, Marseille, France).

### SiRNA knockdown

total of 250,000 A549 or 350,000 HuTu80 cells per well were seeded in 12-well plates 24 h prior to experimentation. Cells were transfected with 15 nM siRNA (AP2M1: Thermo Fisher Scientific #1299001, HSS101955; SCR: Thermo Fisher Scientific #12935300) with 3.33 µL Lipofectamine RNAiMax (Thermo Fisher Scientific, #13778075) following the recommended protocol. The transfection mixture was added dropwise to wells containing 1 mL DPH + 10% FBS and not removed until next day. Cells were split into a six-well plate on the next day. Cells were transfected with 5 nM siRNA (A549) or 15 nM siRNA (HuTu80) with 5 µL Lipofectamine RNAiMax following the recommended protocol. The transfection mixture was added dropwise to wells containing 2 mL DPH + 10% FBS and kept until next day. Cells were seeded into 96-well plates for infection experiment as described above and into 48- or 24-well plates for Western blot. Cells were harvested for Western blotting by addition of 2× loading dye/DTT. Samples were boiled for 10 min and frozen at −20°C. Samples were separated on precast gel (4%–12% NuPAGE Bis-Tris, Thermo Fisher Scientific, #NP0321BOX) with 1× MOPS buffer (Novex, NuPage, #NP0001) for 1 h at 100 V. Proteins were transferred to a nitrocellulose membrane (0.2 µm, BioRad, #162-0112) with transfer buffer (20% methanol, 39 mM glycine, 48 mM Tris base, 0.037% SDS) for 75 min at 100 V. Membranes were blocked with 2% ECL prime blocking reagent in PBS-T (Amersham; #RPN418) and incubated against AP-2 (mouse-anti-AP50, BD, #A611351) and GAPDH (rabbit-anti-GAPDH, Sigma, #G9545-100uL) in blocking buffer over night at 4°C. Signals were detected by chemiluminescence using anti-mouse/rabbit-HRP secondary antibodies (Novex, A16072/A16104) and ECL substrates (Thermo Fisher Scientific, #34577/34095). Membranes were imaged on an Amersham imager 680 (GE/Cytiva).

### Cytotoxicity assays

Cells were prepared as described for the uptake and infectious internalization assays and treated with the respective small molecule inhibitors for 5 h and 24 h, respectively. The viability of cells treated with pitstop2, dynasore, and mβCD was assessed with an MTT [3-(4,5-dimethylthiazol-2-yl)-2,5-diphenyl tetrazolium bromide] assay. MTT reagent (Sigma-Aldrich, #475989–1GM) was resuspended at 5 mg/mL in PBS and diluted at 0.5 mg/mL in warm DMEM. After medium removal, cells were incubated with 50 µL MTT-DMEM for 3 h at 37°C, 5% CO_2_. Thereafter, the MTT reagent was removed, and purple crystals within the cells were resuspended by addition of 50 µL DMSO. Absorbance was measured at 570 nm and 630 nm (reference wavelength) on a Multiskan Go (Thermo Fisher Scientific). The viability after cytoD treatment was assessed with CellTiter-Glo (Promega, #G7570). Cells ad reagents were equilibrated to room temperature. Reconstituted CellTiter-Glo reagent was added at a 1:1 ratio to the treated wells, mixed by pipetting for cell lysis, and incubated for 30 min at room temperature. Luciferase signal was measured on a Berthold luminometer with the software MikroWin, version 5.22.

### Cell surface receptor staining

Cells were detached with PBS/EDTA (0.05%), pelleted, suspended in DPH + 10% FBS, and kept in suspension on a shaker for 1 h at 37°C for recovery. A total of 2 × 10^5^ cells per well were pelleted in a v-bottom 96-well plate and washed with cold PBS + 2% FBS. Antibodies against CAR (clone RmcB, Merck, #05-644), HS (clone F58-10E4, amsbio, #370255S), alpha2 (clone P1E6, Merck, MAB1950Z), alpha3 (clone ASC-1, Merck, MAB2056), alphaV (clone P2W7, Abcam, ab11470), and alpha6 (clone MP4F10, Abcam, ab20142) were diluted in cold PBS + 2% FBS to a working concentration of 0.5–1 µg/mL. Cells were incubated with 100 µL antibody dilution for 30 min on ice in shaking conditions. Cells were pelleted, resuspended in PBS, pelleted again, and incubated with 100 µL secondary antibody dilution (goat anti-mouse IgG, IgM Alexa Fluor 488, Thermo Fisher Scientific, #A-10680) at a working concentration of 1 µg/mL in PBS + 2% FBS for 30 min on ice in shaking conditions. Cells were washed again and suspended in PBS + 2% FBS as above. Cell surface staining was measured by flow cytometry in a Bio-Rad ZE5 and analyzed with FlowJo version 10.

### Transmission electron microscopy

A total of 1 × 10^6^ A549 and HuTu80 cells per well were seeded in a six-well plate on the day prior to experimentation. Cells were transferred to ice and washed twice with cold DPH. Viruses were diluted at 50,000 particles per cell [based on 11 ng virus preparation contains 4 × 10^7^ particles (64)] and added to cells on ice. Samples were incubated on ice for binding for 1 h shaking. Thereafter, virus inoculum was removed and warm DPH + 2% FBS was added to each well. Samples were transferred to 37°C, and internalization was allowed for 12.5 min, 2 h, and 6 h. After incubation, medium was removed, and 1.5 mL EM fixative [2.5% glutaraldehyde, 0.05% malachite green, 0.1 M phosphate buffer (PB; NaH_2_PO_4_ + MQ-water)] (Taab Laboratory Equipment, Ltd., Aldermaston, UK) for 1 h at room temperature. Fixed samples were washed twice with 2 mL 0.1 M PB and kept in 0.1 M PB until processing. All the below chemical fixation steps were performed in the microwave Pelco biowave pro+ (Ted Pella, Redding, CA) unless stated. The samples were rinsed 2× with PB and post-fixed with 0.8% K_3_Fe(CN)_6_ and 1% OsO_4_ in 0.1 M PB for 14 min and were rinsed 2× with MQ-water. The samples were then stained with 1% aqueous tannic acid. Following 2× rinse in MQ-water, samples were stained with 1% aqueous uranyl acetate (Polysciences, Inc., Hirschberg an der Bergstrasse, DE). After 2× MQ-water rinses, samples were dehydrated in gradients of ethanol (30%, 50%, 70%, 80%, 90%, 95%, 100%, and 100%). The samples were infiltrated with graded series of Spurr resin in ethanol (1:3, 1:1 and 3:1). After microwave, samples were infiltrated twice with 100% resin before polymerization overnight at 60°C. Samples were sectioned (70 nm) and imaged with Talos 120C (FEI, Eindhoven, The Netherlands) operating at 120 kV at the Umeå Core Facility for Electron Microscopy. Micrographs were acquired with a Ceta 16M CCD camera (FEI, Eindhoven, The Netherlands).

### Diffusion measurement

Imaging of AdV particles diffusing on live cells was performed using a Nikon Ti2-E inverted microscope equipped with a two-color laser source (wavelengths: 488 nm and 562 nm), a Prime 95B sCMOS camera (Teledyne Photometrics), and a multiband filter cube (86012v2 DAPI/FITC/TxRed/Cy5, Nikon Corp.). The images were collected using a 60× oil immersion objective (numerical aperture: 1.49, MRD01691, Nikon Corp.), 488 nm excitation wavelength, and HILO microscopy configuration, which allows imaging of the apical cell surface while reducing out-of-focus signal and background noise when compared to epifluorescence. For the experiments, 40,000 A549 and 50,000 HuTu80 cells per well were seeded in a glass-bottom 96-well plate 24 h before imaging. The following day, the culture medium was removed and replaced with warm DMEM without phenol red and 2% FBS to reduce the background signal in fluorescence imaging. Cells were incubated at 37°C for 1 h, and, right before the experiment, the plate was transferred under the microscope and kept at 37°C and 100% humidity in a dedicated on-stage incubator (Okolab). One hundred microliters of AF488-labeled HAdV in a premixed 1:50 dilution were added in each well, pipetting thoroughly to homogenize the virus particle distribution in the cell medium. After 5 min, the cell supernatant was removed, and the well was washed twice with fresh prewarmed medium. Cells were imaged 5 and 30 min after washing. Videos were acquired at 10 frames per second for 2 min. Two videos were acquired for the 5 min and between 2 and 4 videos for the 30 min time point. The frame rate was optimized to guarantee good time resolution and a high signal-to-noise ratio. All conditions were acquired in duplicates.

### Single-particle tracking and diffusion analysis

The recorded time lapses were processed to remove the background and improve contrast with an in-house ImageJ script. First, a Gaussian filter with a sigma of 0.5 pixels was convolved to the image to reduce the camera noise. Bleaching was then corrected using a built-in Fiji function, which fits the variation of total intensity in time to an exponential fit (63). The background signal was calculated by averaging over all the frames and then applying a Gaussian filter with a sigma of 20 pixels. This removes the fine details, like immobile viruses, while maintaining the broader cellular features. The background was then subtracted from each frame. The virus particles were then detected and tracked using the Laplacian Gaussian filter in the TrackMate plugin, which allows for sub-pixel resolution on the particle position (65). Linking between frames is performed using a maximum linking displacement of 1 µm and a maximum frame gap of 10. The obtained trajectories were segmented according to the characteristics of the motion displayed using a DC-MSS algorithm (28), and each segment was assigned to a motion type, according to the slope of the moment scaling spectrum. The motion types considered were immobile, confined motion, free diffusion, and directed motion. In-house scripts were used to extract diffusion parameters, e.g., diffusion coefficient (D) and confinement radius (R_C_), of both each segment and each unsegmented track. Only tracks with a duration of more than 300 frames (30 s) were considered in the analysis of unsegmented tracks. The tracks were classified considering the average time spent in each motion type. A numerical value was assigned to each motion type (0, immobile; 1, confined motion; 2, free diffusion; 3, directed motion), and the weighted average over the time spent in each motion type was calculated resulting in a numerical coefficient (“average motion coefficient”) between 0 (fully immobile) and 3 (only directed motion). A value of 1.5 was considered as the threshold between particles dominated by confined diffusion and particles displaying mostly free or directed motion.

### Fiber knob proteins

DNA isolation from HAdV-C5, HAdV-F40, and HAdV-F41 virions was performed using a Blood & Cell Culture DNA minikit (Qiagen). Templates for the fiber knobs of C5 (amino acids 387 to 581), F40 (amino acids 348 to 547), and F41 (amino acids 363 to 562) were amplified from viral DNA by PCR. Fragments were then cloned into a pQE30Xa expression vector encoding an N-terminal His tag (Qiagen) using restriction sites for BamHI and HindIII (Fermentas, Thermo Fisher Scientific). Proteins were expressed in *Escherichia coli* strain M15 and purified with nickel-nitrilotriacetic acid agarose beads according to the manufacturer’s protocol (Qiagen). Proteins were analyzed by denaturing gel (NuPAGE Bis-Tris; Invitrogen, Life Technologies) and Western blotting using monoclonal antibodies (MAbs) against the His tag (RGS-His mouse monoclonal, Qiagen, #34650).

### Surface plasmon resonance analyses

All measurements of HAdV long fiber knobs:CAR interactions were performed at 25°C using a Biacore T-200 instrument. CAR (CXADR Fc chimera full-length extracellular D1D2 domain; R&D Systems, Minneapolis, MN, USA) was immobilized to a CM5 chip, using the Amine Coupling Kit (GE Healthcare), to a concentration of 1.2 to 1.5 ng·mm^2^ (∼1,500 response units). All binding assays were performed at 25°C using running buffer [10 mM phosphate buffer (pH 7.4), 140 mM NaCl, 0.27 mM KCl, and 0.05% Tween20]. The analytes were diluted in running buffer and serially diluted in running buffer in a twofold concentration dilution (2000 to 7.81125 nM) and then injected in series over the reference and experimental biosensor surfaces for 120 s at a flow rate of 30 µL/min. Blank samples containing only running buffer were also injected under the same conditions to allow for double referencing. After each cycle, the biosensor surface was regenerated with a 60-s pulse of 10 mM glycine (pH 1.5) at a flow rate of 30 µL/min.

### Software

Graphs and statistics were generated with GraphPad Prism 10, version 10.0.2. Schemes were created with BioRender (biorender.com). Figures were assembled with Adobe Illustrator version 27.8.1.
